# Use of larvae of the wax moth *Galleria mellonella* as an in vivo model to study the virulence of *Helicobacter pylori*

**DOI:** 10.1186/s12866-014-0228-0

**Published:** 2014-08-27

**Authors:** Maria Giannouli, Anna Teresa Palatucci, Valentina Rubino, Giuseppina Ruggiero, Marco Romano, Maria Triassi, Vittorio Ricci, Raffaele Zarrilli

**Affiliations:** Department of Molecular Medicine, Human Physiology Section, University of Pavia Medical School, Pavia, Italy; Department of Public Health, Hygiene Section, University of Naples “Federico II”, Naples, Italy; Department of Translational Medical Sciences, University of Naples “Federico II”, Naples, Italy; PhD School of Science, University of Basilicata, Potenza, Italy; Department of Clinical and Experimental Medicine, Chair of Gastroenterology, Second University of Naples, Naples, Italy; CEINGE Biotecnologie Avanzate, Naples, Italy

**Keywords:** *Helicobacter pylori*, Virulence factors, *Galleria mellonella*, Infection, Apoptosis

## Abstract

**Background:**

*Helicobacter pylori* is the first bacterium formally recognized as a carcinogen and is one of the most successful human pathogens, as over half of the world’s population is colonized by the bacterium. *H. pylori*-induced gastroduodenal disease depends on the inflammatory response of the host and on the production of specific bacterial virulence factors. The study of *Helicobacter pylori* pathogenic action would greatly benefit by easy-to-use models of infection.

**Results:**

In the present study, we examined the effectiveness of the larvae of the wax moth *Galleria mellonella* as a new model for *H. pylori* infection. *G. mellonella* larvae were inoculated with bacterial suspensions or broth culture filtrates from either different wild-type *H. pylori* strains or their mutants defective in specific virulence determinants, such as VacA, CagA, CagE, the whole pathogenicity island (PAI) *cag*, urease, and gamma-glutamyl transpeptidase (GGT). We also tested purified VacA cytotoxin. Survival curves were plotted using the Kaplan-Meier method and LD_50_ lethal doses were calculated. Viable bacteria in the hemocoel were counted at different time points post-infection, while apoptosis in larval hemocytes was evaluated by annexin V staining. We found that wild-type and mutant *H. pylori* strains were able to survive and replicate in *G. mellonella* larvae which underwent death rapidly after infection. *H. pylori* mutant strains defective in either VacA, or CagA, or CagE, or *cag* PAI, or urease, but not GGT-defective mutants, were less virulent than the respective parental strain. Broth culture filtrates from wild-type strains G27 and 60190 and their mutants replicated the effects observed using their respective bacterial suspension. Also, purified VacA cytotoxin was able to kill the larvae. The killing of larvae always correlated with the induction of apoptosis in hemocytes.

**Conclusions:**

*G. mellonella* larvae are susceptible to *H. pylori* infection and may represent an easy to use in vivo model to identify virulence factors and pathogenic mechanisms of *H. pylori*. The experimental model described can be useful to screen a large number of clinical *H. pylori* strain and to correlate virulence of *H. pylori* strains with patients’ disease status.

## Background

*Helicobacter pylori* is a gram-negative, microaerophilic bacterium that colonizes approximately 50% of the world’s population. *H. pylori* infection causes chronic gastritis, which is asymptomatic in the majority of carriers but may evolve into more severe disease, such as atrophic gastritis, gastric and duodenal ulcers, mucosa-associated lymphoid tissue lymphoma and gastric adenocarcinoma [1,2]. *H. pylori*-induced gastroduodenal disease depends on the inflammatory response of the host and on the production of specific bacterial virulence factors, such as urease, the vacuolating cytotoxin VacA, gamma-glutamyl transpeptidase (GGT), and a 40-kbp pathogenicity island (*cag* PAI) encoding the 120–145 kDa immunodominant protein cytotoxin-associated gene A (CagA) as well as a type IV secretion system that injects CagA into the host cell [1–9]. The availability of a large number of genome sequences of *H. pylori* strains isolated from asymptomatic individuals and patients with gastric cancer, peptic ulcer disease, or gastritis provides the opportunity to identify novel virulence factors and mechanisms of diseases [10–12].

In vitro models of monolayers of gastric mucosal cells, either primary cultures or gastric adenocarcinoma cell lines, primary cultures of monocytes or lymphocytes have been extensively used to study *H. pylori* pathogenesis but have not been able to reproduce completely clinical outcomes associated with *H. pylori* infection [6,13–15]. Moreover, rodent models of wild-type mice, knock-out or transgenic mice and mongolian gerbils have been used to reproduce *H. pylori* persistent infection and disease [16–18]. However, these mammalian models are very expensive and time-consuming because they require specific animal facilities not widely accessible to all research groups, a large number of animals in order to obtain statistically significant results, and a formal approval by the local Ethics Committee.

Invertebrate hosts, such as nematodes or insects, can be used as alternative models of infection. *Caenorhabditis elegans* has been used as an infection model for a diverse range of bacterial and fungal pathogens [19,20]. However, *C. elegans* cannot survive at 37°C and lacks functional homologues of cellular components of the mammalian immune system, such as specialized phagocytic cells [21]. Models of infection based on insects, such as *Drosophila melanogaster* and *Galleria mellonella* (wax moth) larvae offer the advantage that they can survive at 37°C. For example, a transgenic *Drosophila* model with inducible CagA expression has been used to study the signal transduction pathways activated by CagA [22,23]. In addition, insects possess specialized phagocytic cells, also known as hemocytes [21], which resemble mammalian phagocytes because they are able to engulf pathogens and kill them by using antimicrobial peptides and reactive oxygen species through proteins homologous to the NADPH oxidase complex of human neutrophils [24]. Moreover, genes that are known to mediate recognition of pathogen-associated molecular patterns, such as at least three different toll-like receptors and the transcription factor nuclear factor-κB (NFkB), and apoptosis-related signaling, such as caspases-1, −3,-4, and −6, are expressed in *G. mellonella* larvae [25,26]. Although *G. mellonella* does not reproduce all aspects of mammalian infection, their larvae are increasingly used as mini-hosts to study pathogenesis and virulence factors of several bacterial and fungal human pathogen for the following advantages: i) low overall costs of breeding large numbers of larvae and worldwide commercial availability; ii) adaptation to human physiological temperature (37°C); iii) presence of a well-characterized phagocytic system; iv) availability of a comprehensive transcriptome and immune gene repertoire [21,24–26]. *G. mellonella* larvae were shown to be susceptible to infection with several pathogens, including *Pseudomonas aeruginosa* [27], *Staphylococcus aureus* [28], *Francisella tularensis* [29], *Burkholderia mallei* [30], *Acinetobacter baumannii* [31], *Klebsiella pneumoniae* [32], *Cryptococcus neoformans* [33], *Candida albicans* [34] and *Campylobacter jejuni* [35]. Also, *C. jejuni* bacteria have been observed in the haemocoel and gut of infected larvae, and have been demonstrated to induce damage to the midgut [36].

In this study, we demonstrate that *G. mellonella* is susceptible to infection with *H. pylori* and may represent a valuable model to identify virulence factors and pathogenic mechanisms of *H. pylori*.

## Methods

### Bacterial strains and growth conditions

A total of eleven *H. pylori* strains were included in this study. In particular, we used: a) the wild-type *H. pylori* strain G27 (VacA^+^/*cag*PAI^+^/urease^+^) and its isogenic mutants in which the *cagA* (G27Δ*cagA)* or *cagE* (G27Δ*cagE*) gene or the entire *cag*PAI (G27Δ*cagPAI*) were disrupted by insertional mutagenesis [3,37]; b) the wild-type *H. pylori* strain 60190 (ATCC 49503; VacA + s1/i1/m1/*cag*PAI^+^/urease^+^) and its isogenic mutants in which *vacA* (60190Δ*vacA*), or *cagA* (60190Δ*cagA*), or *cagE* (60190Δ*cagE*) were disrupted by insertional mutagenesis [38,39] as well as its urease-negative spontaneous mutant urease (60190 Urease-negative) [40]; c) the mouse-adapted *H. pylori* strain M5 and its GGT-defective isogenic mutant (M5*ggt::aph*) in which *ggt* was disrupted by insertional mutagenesis [8].

Bacteria were cultured on Columbia agar supplemented with 10% defibrinated horse blood, 1% Vitox and Skirrow’s supplement under microaerophilic conditions in anaerobic jars with microaerobic System CampyGen (all from Oxoid, Milan, Italy) at 37°C for 3 days.

### Preparation of broth culture filtrates (BCFs)

BCFs were prepared as previously described [41,42]. Briefly, bacteria were grown in Brucella broth medium supplemented with 1% Vitox and Skirrow as well as 5% heat-inactivated fetal calf serum (FCS; Sigma-Aldrich, Milan, Italy) in anaerobic jars with microaerobic System CampyGen with gentle shaking (150 oscillations/min) for 24–48 h at 37°C. When bacterial suspensions reached 1.0 optical density units at 450 nm (corresponding to a bacterial concentration of 5 × 10^8^ colony-forming units (CFUs/ml), bacteria were removed by centrifugation (12,000 *g* for 15 min), and the supernatants were sterilized by filtering through a 0.22-μm-pore-size cellulose acetate filter (Sartorius Minisart SM 16534, Sigma-Aldrich) to obtain BCFs.

### Purification and use of VacA toxin

VacA (s1/m1 genotype) was purified by ammonium sulphate precipitation and gel filtration chromatography from wild-type *H. pylori* 60190 strain grown in Brucella broth in which foetal calf serum was replaced by 0.2% β-cyclodextrins (Sigma-Aldrich) [43,44]. Purified VacA was stored in melting ice and, immediately before use on *G. mellonella* larvae, was activated or not by dropwise acidification to pH 3.0 with 0.2 N HCl. Vacuolating activity of purified VacA was determined by means of neutral red uptake as previously described [45]. In order to compare the effects obtained with either purified VacA or VacA^+^ BCF, it must be taken into account that the vacuolating power on cultured human epithelial cells of VacA^+^ BCF prepared as above from 60190 *H. pylori* strain was equivalent to that exhibited by a final concentration of 1.2 μg/ml of activated purified VacA [42,45].

### *G. mellonella* killing assays

To assess the virulence of *H. pylori* in vivo using the *G. mellonella* insect model of infection [26], caterpillars weighing between 200 mg and 400 mg and maintained on wood chips in the dark at 8-10°C were employed in all assays. No ethical approval was required for the study because there was no use of a mammalian model of infection and animal house. Briefly, bacteria were harvested from a culture by rolling a moistened swab over the plate into 1 ml of phosphate-buffered saline (PBS) and adjusted to an OD_450_ of 1.0. A Hamilton syringe was used to inject 10 μl aliquots of serially diluted bacterial suspensions (from 1 × 10^7^ to 1 × 10^4^ CFUs) or BCFs collected from 1 × 10^6^ CFUs into the hemocoel via the left proleg of each larva. Bacterial colony counts on 10% blood Columbia agar plates under microaerophilic conditions were used to confirm all inocula of either bacterial suspensions or BCFs. Control larvae were either injected with 10 μl of PBS in order to measure any potential lethal effects of the injection process, or not injected to measure the effects of the incubation procedure. Ten *G. mellonella* larvae were infected for each experimental condition, with each experiment repeated at least 3 times. After injection, larvae were incubated in petri dishes at 37°C in standard aerobic conditions and survival was recorded at 24 h intervals for 96 h. Larvae were considered dead when they displayed no movement in response to gentle prodding with a pipette tip [31].

To determine the numbers of viable bacteria in larvae at 0, 24, 48 and 72 h post-infection, larvae were chilled on ice for 10 min. The bottom 2 mm of each larva was aseptically removed and haemocoel was drained into a sterile 1.5 ml microcentrifuge tube. For enumeration haemocoel was serially diluted in PBS and the bacterial load per larva was quantified by enumeration of CFUs on Columbia Blood Agar plates (CBA) supplemented with 10% defibrinated horse blood, 1% Vitox and Skirrow’s supplement and incubating under microaerophilic conditions in anaerobic jars with microaerobic System CampyGen (Oxoid) at 37°C for 48-72 h.

### Flow cytometry analysis of *G. mellonella* hemocytes

Hemocytes were prepared from hemolymph of *G. mellonella* larvae as described by Bergin et al. [24]. Plasma membrane asymmetry existing in living cells is lost on apoptosis and it is commonly detected with probes, like Annexin V, interacting strongly and specifically with phosphatidylserine. In order to assess apoptosis induction on *G. mellonella* hemocytes, (FITC)-conjugated annexin V (Pharmingen San Diego, CA) staining has been performed as described [46]. Cells were washed in cold Annexin V buffer (10 mM HEPES, 140 mM NaCl, 2.5 mM CaCl_2_) prior to treatment with FITC-labeled Annexin V (BD, Milan, Italy) for 15 min at room temperature. Annexin V binding was evaluated by using a two laser equipped FACSCalibur apparatus and the Cell Quest analysis software (Becton Dickinson, Mountain View, CA).

### Statistical analyses

All statistical analyses were carried out using GraphPad Prism version 5.04 for Windows (GraphPad Software, San Diego, CA, USA). Survival curves were plotted using the Kaplan-Meier method, and differences in survival were calculated using the log-rank test for multiple comparisons. Differences were considered statistically significant at *P* < .05. LD_50_ values of *H. pylori* strains were calculated as described previously [47]. Briefly, GraphPad Prism was used to fit a curve to the infection data of the following form: *Y* = [*A* + (1 − *A*)]/[1 + exp(*B* − *G*xlnX)], where X is the number of viable bacterial cells injected, Y the fraction of larvae killed by the bacterial solution, A is the fraction of larvae killed by the control solution, and B and G are curve-fitting constants automatically calculated by GraphPad Prism. LD_50_ was calculated as the value of X that corresponds to Y = 0.5. All experiments were performed at least three times and the results were shown as means ± SEM. Differences between mean values were tested for significance by performing either unpaired, two-tailed Student’s *t*-tests or one-way ANOVA analysis followed by Tukey’s multiple-comparison test, when appropriate. A *P* value <0.05 was considered to be statistically significant.

## Results

### *H. pylori* infection causes death of *G. mellonella* larvae

**Figure 1:**
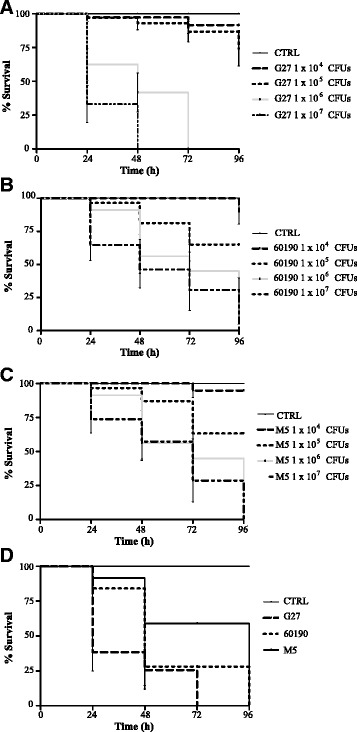
**Survival of** ***G. mellonella*** **following infection by** ***H. pylori*** **strains.** Kaplan-Meier survival curves of *G. mellonella* larvae after 24 h-96 h from injection with 1 × 10^4^, 1 × 10^5^, 1 × 10^6^ and 1 × 10^7^ CFUs of wild type strains G27 (panel **A**), 60190 (panel **B**), M5 (panel **C**) are shown. Kaplan-Meier survival curves of *G. mellonella* larvae after 24 h-96 h from injection with 1 × 10^6^ CFUs of wild-type *H. pylori* strains G27, 60190 and M5 (panel **D**) are shown. The data shown are means ± SEM from three independent experiments recorded for 96 h. Differences in survival were calculated using the log-rank test for multiple comparisons. Differences were considered statistically significant at *P* < 0.05. PBS, phosphate-buffered saline.

We examined the susceptibility of *G. mellonella* to wild-type *H. pylori* strains G27, 60190 and M5, which are widely used for molecular pathogenesis studies. *G. mellonella* larvae were injected with 1 × 10^4^, 1 × 10^5^, 1 × 10^6^ and 1 × 10^7^ CFUs of G27 and 60190 wild-type strains and incubated at 37°C up to 96 h. As shown in Figure 1A, 1B and 1C, *H pylori* strains G27, 60190 and M5 caused a time- and dose-dependent death of larvae (p < 0.0001). The percentage of surviving larvae at 24 h after infection with increasing doses of wild-type strains G27, 60190 and M5 ranged between 97% and 33%, 100% and 65%, and 100% and 74%, respectively. No mortality was observed in *G. mellonella* larvae either non-infected or PBS-injected (Figure 1A, 1B, 1C). Since the dose of 1 × 10^6^ CFUs/larva allowed to observe clear-cut differences in virulence potential, this concentration of bacterial suspension was chosen as the optimal dosage for the subsequent virulence studies described in this paper. As shown in Figure 1D, wild-type strain G27 showed significantly increased mortality compared to wild-type strains 60190, and M5 (*P <*0.0005). Separately, the 50% lethal doses (LD_50_) of G27, 60190, and M5 were determined in *G. mellonella*. The analysis of LD_50_ doses of *H. pylori* wild-type strains tested showed that G27 was more virulent than 60190 and M5, with LD_50_ values at 48 h of 2.78 ± 0.4, 6.1 ± 0.4 and 12.8 ± 0.3 × 10^5^ CFUs, respectively (Table 1). Collectively, these results demonstrate that *G. mellonella* is susceptible to *H. pylori* infection, in a time- and dose-dependent manner.

**Table 1:** **Lethal dose 50**% **of** ***H. pylori*** **strains in** ***Galleria mellonella***

	**LD**_**50**_ **(means ± SEM)** ^*^	
**Strains**	**48 h**	**72 h**
G27	2.8 (±0.4) × 10^5^	2.4 (±0.2) × 10^5^
G27Δ*cag*A		3.1 (±0.04) × 10^6^
G27Δ*cag*E		2.4 (±0.06) × 10^6^
G27Δ*cagPAI*		2.0 (±0.01) × 10^6^
60190	6.1 (±0.4) × 10^5^	1.4 (±0.04) × 10^6^
60190Δ*vacA*		8.2 (±0.04) × 10^6^
60190Δ*cagA*		9.7 (±0.04) × 10^6^
60190Δ*cagE*		9.5 (±0.06) × 10^6^
60190Urease-negative		8.7 (±0.04) × 10^6^
M5	12.8 (±0.3) × 10^5^	2.1 (±0.08) × 10^5^
M5 *ggt::aph*	12.0 (±0.6) × 10^5^	1.0 (±0.1) × 10^5^

^*^The LD_50_ values were expressed in Colony Forming Units (CFUs).

### Effect of *H. pylori* virulence factors on killing of *G. mellonella* larvae

**Figure 2:**
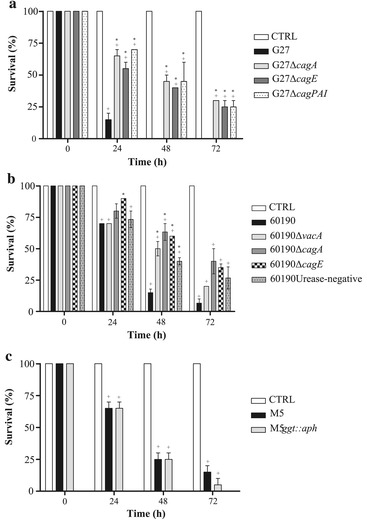
**Ability of bacterial suspensions of 1 × 10** ^**6**^ **CFUs wild-type strain G27 and their mutants (panel A), wild type strain 60190 and their mutants (panel B) and wild type strain M5 and** ***ggt*** **-mutant (panel C) to kill** ***G. mellonella*** **larvae at different time points.** Values represent the mean (±SEM) of three independent experiments. + *P* < 0.05 vs control (ANOVA).*; * *P* < 0.05 vs wild-type strain (ANOVA). CTRL, control.

To identify bacterial virulence factors responsible for *H. pylori*-induced killing of *G. mellonella* larvae, we compared the effects of wild-type strains G27, 60190 and M5 with those of their respective mutants in selective virulence factors. The survival percentages of a group of 10 *G. mellonella* larvae during 72 h post-infection with 1 × 10^6^ CFUs of bacterial suspension were analyzed. As shown in Figure 2A, the wild-type strain G27 showed a statistically significant higher virulence compared with G27Δ*cagPAI*, (i.e., the G27 isogenic mutant in which the entire *cag* PAI has been deleted), or G27Δ*cagA, or* G27Δ*cagE* (i.e., the G27 isogenic mutants in the effector protein CagA or in the regulatory protein CagE of the type IV secretion system, respectively). Indeed, we found 15% of larvae and no larvae alive after respectively 24 h and 48 h infection with wild type G27 strain, while 55%-70% and 40-45% of larvae alive after 24 h and 48 h infection with mutant strains. Moreover, the wild-type strain 60190 showed a statistically significant increased virulence compared with its isogenic mutants defective in either CagA, or CagE, or VacA as well as with its spontaneous mutant defective in urease at 48 h (Figure 2B). In contrast, there was no significant difference between wild type strain M5 and its GGT-defective isogenic mutant M5 *ggt::aph* at any time post-infection (Figure 2C).

Concordantly, LD_50_ values of *H. pylori* mutant strains defective in either VacA, or CagA, or CagE, *cag* PAI or urease- but not GGT-defective mutant, exhibited slower killing action than their respective wild type strains (Table 1). Also, all wild type strains G27, 60190 and M5 and their respective mutant strains showed a statistically significant effect on killing of *G. mellonella* larvae compared with control non-infected larvae (p < 0.05) (Figure 2A-C).

Taken together, the data shown indicate that killing of larvae by *H. pylori* was at least in part dependent on the expression of a functional *cag* PAI, CagA, VacA cytotoxin and urease but independent of GGT.

We next determined whether death of *G. mellonella* was associated with the growth of *H. pylori* wild-type and mutant strains in the infected larvae. The larvae were injected with 1 × 10^6^ CFUs of *H. pylori* strains as described above and the number of viable bacteria within the hemolymph of *G. mellonella* infected larvae was determined after every 24 h interval. As shown in Table 2, wild-type and mutant *H. pylori* strains showed similar time-dependent increases of 1-log in the number of bacteria with no significant differences observed among strains (*P >*0.05). The above data suggest that *H. pylori* is able to replicate in *G. mellonella* larvae independently of the strain virulence and that differences in killing observed between wild-type strains and mutants are not due to impaired ability of mutants to replicate into the infected host.

**Table 2:** **Viable count (CFUs; means ± SEM) of** ***H. pylori*** **strains in** ***G. mellonella*** **at 24, 48 and 72 h post-infection**

**Strains**	**T0**	**24 h**	**48 h**	**72 h**
G27	1.1 (±0.06) × 10^6^	3.9 (±0.03) × 10^6^	5.2 (±0.8) × 10^6^	1.6 (±0.3) × 10^7^
G27Δ*cag*A	1.6 (±0.2) × 10^6^	2.8 (±0.06) × 10^6^	4.6 (±0.4) × 10^6^	1.1 (±0.2) × 10^7^
G27Δ*cag*E	1.0 (±0.1) × 10^6^	2.2 (±0.04) × 10^6^	4.0 (±0.6) × 10^6^	9.2 (±0.3) × 10^6^
G27Δ*cagPAI*	1.2 (±0.3) × 10^6^	2.0 (±0.02) × 10^6^	3.6 (±0.4) × 10^6^	8.6 (±0.2) × 10^6^
60190	1.6 (±0.1) × 10^6^	5.2 (±0.02) × 10^6^	7.8 (±0.1) × 10^6^	1.8 (±0.9) × 10^7^
60190Δ*vacA*	8.4 (±0.2) × 10^5^	1.9 (±0.04) × 10^6^	3.9 (±0.1) × 10^6^	9.4 (±0.3) × 10^6^
60190Δ*cagA*	1.2 (±0.1) × 10^6^	2.1 (±0.05) × 10^6^	4.2 (±0.2) × 10^6^	1.2 (±0.3) × 10^7^
60190Δ*cagE*	1.0 (±0.04) × 10^6^	1.8 (±0.03) × 10^6^	3.4 (±0.4) × 10^6^	1.0 (±0.3) × 10^7^
60190Urease-negative	1.4 (±0.06) × 10^6^	2.6 (±0.2) × 10^6^	4.9 (±0.4) × 10^6^	9.8 (±0.2) × 10^6^
M5	1.3 (±0.04) × 10^6^	2.0 (±0.4) × 10^6^	4.2 (±0.5) × 10^6^	1.2 (±0.2) × 10^7^
M5*ggt::aph*	1.2 (±0.04) × 10^6^	1.8 (±0.2) × 10^6^	3.6 (±0.6) × 10^6^	9.6 (±0.4) × 10^6^

The number of viable bacteria in infected larvae were determined as described in the Methods section and expressed in CFUs.

### Effect of BCFs from different wild-type *H. pylori* strains or their isogenic mutants and of purified VacA toxin on killing of *G. mellonella* larvae

**Figure 3:**
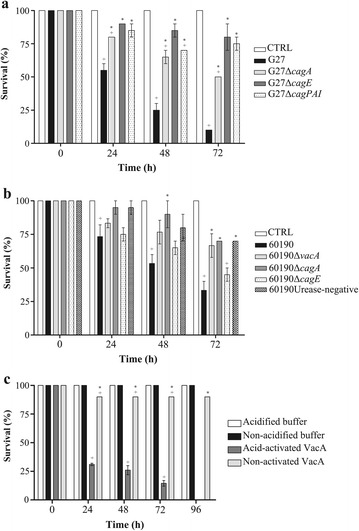
**Ability of broth culture filtrates from 1 × 10** ^**6**^ **CFUs wild-type strain G27 and their mutants (panel A), wild type strain 60190 and their mutants (panel B) and VacA cytotoxin (panel C) to kill** ***G. mellonella*** **larvae at different time points.** Values represent the mean (±SEM) of three independent experiments. + *P* < 0.05 vs control (ANOVA); * *P* < 0.05 vs wild-type strain (ANOVA). CTRL, control.

To evaluate whether the killing of *G. mellonella* larvae by *H. pylori* was dependent on a soluble bacterial virulence factor(s), the effect of BCFs from G27, 60190 and their mutants and purified VacA on killing of *G. mellonella* larvae was investigated. As shown in Figure 3A and 3B, BCFs from wild-type strains G27 and 60190 strains caused a time-dependent death of *G. mellonella* larvae with 10% and 35% of survival after 72 h of injection, respectively. Also, BCFs from wild-type strain G27 induced statistically higher killing of *G. mellonella* larvae than G27Δ*cagPAI*, G27Δ*cagA* and G27Δ*cagE* isogenic mutant strains at 24 h, 48 h and 72 h post injection respectively; similarly, BCFs from wild-type strain 60190 induced higher killing of larvae than 60190Δ*cagA* at 48 h and 72 h, and 60190Urease-negative mutant at 72 h post-injection. No mortality was observed in the *G. mellonella* larvae injected with uninoculated broth filtrate taken as a control (Figure 3A and 3B). Moreover, injection of acid-activated VacA cytotoxin from 60190 *H. pylori* strain caused time-dependent death of larvae, with 31% survival at 24 h post-injection and no larvae alive at 96 h post-injection. On the contrary, injection of non-activated VacA caused death of 10% of larvae, injection of acidified or non-acidified control buffers caused no deaths of larvae (Figure 3C). These data indicate that the effect of *H. pylori* on killing of larvae is mediated at least in part by bacterial soluble virulence factors, including VacA cytotoxin, CagA and *cag* PAI-encoded proteins.

### *H. pylori* G27 and 60190 and their isogenic mutants, BCFs and VacA induce apoptosis of *G. mellonella* hemocytes

**Figure 4:**
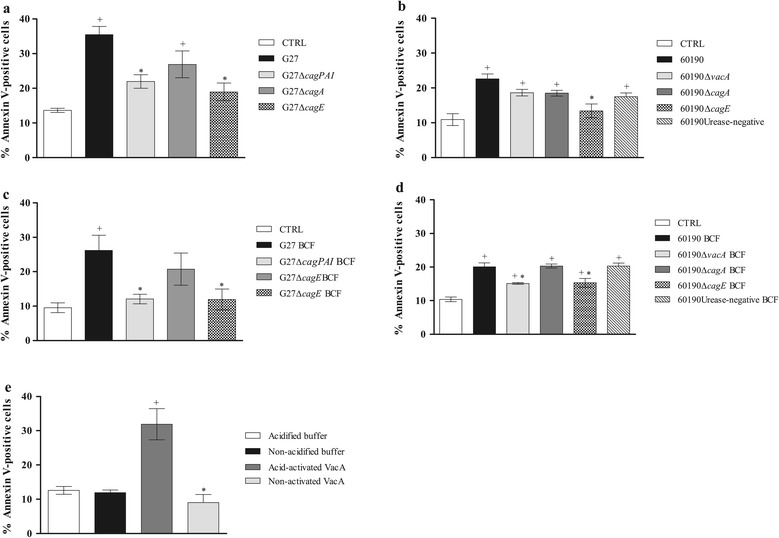
**Determination of Annexin V binding on hemocytes from** ***G. mellonella*** **larvae injected with** ***H. pylori*** **bacteria suspensions, BCFs or purified VacA cytotoxin.** Percentage of Annexin V-positive hemocytes of *G. mellonella* larvae after 3 h from injection with bacterial suspensions of wild-type strain G27 and their mutants (panel **A**), bacterial suspensions of wild-type 60190 and their mutants (panel **B**), BCFs of wild-type strain G27 and their mutants (panel **C**), BCFs from wild-type 60190 and their mutants (panel **D**) and purified VacA cytotoxin (panel **E**). As control, Annexin V binding on non-treated hemocytes was always performed. Values represent the mean (±SEM) of three independent experiments. + *P* < 0.05 vs control (ANOVA);* *P* < 0.05 vs wild-type strain (ANOVA). CTRL, control; BCF, broth culture filtrate.

Because it has been shown that *H. pylori* triggers the apoptotic program in different experimental systems [2,7,9,14,23,48], we evaluated whether the killing of *G. mellonella* larvae by *H. pylori* might be mediated also through induction of apoptosis. To address this issue, we evaluated annexin V binding on hemocytes from *G. mellonella* larvae injected with bacterial suspension or BCFs of wild-type strains and mutants or purified VacA cytotoxin. As control, annexin V binding on uninfected hemocytes was analyzed. As shown in Figure 4A, *H. pylori* wild type strain G27 increased annexin V staining in *G. mellonella* hemocytes by 3.5-fold compared with control uninfected larvae, while G27*ΔcagE* and G27*ΔcagPAI* increased annexin V staining by approximately 2-fold (p < 0.05 vs G27 strain). Concordantly, *H. pylori* wild type strain 60190 increased annexin V staining in *G. mellonella* hemocytes by approximately 2.5-fold, while the 60190Δ*cagE* demonstrated a significantly less capacity to bind the annexin (Figure 4B). These data indicate that *H. pylori* induction of apoptosis in *G. mellonella* hemocytes is at least in part dependent on the expression of genes in the *cag* PAI.

We next evaluated the effect of soluble virulence factor(s) on apoptosis in *G. mellonella* hemocytes. As shown in Figure 4C, BCFs from G27 increased annexin staining by 2.5-fold, while BCFs from G27Δ*cagE* and G27Δ*cagPAI* demonstrated a significantly lower capacity to bind the annexin compared with BCFs from G27 strain (p < 0.05). Also, BCFs from *H. pylori* wild type strain 60190 increased annexin V staining in *G. mellonella* hemocytes by approximately 2-fold, while the 60190*ΔvacA* and 60190*ΔcagE* mutants demonstrated a significantly lower capacity to bind the annexin compared with BCFs from 60190 strain (*P <*0.05) (Figure 4D). Moreover, activated VacA increased annexin V staining of *G. mellonella* hemocytes by 3-fold compared with non-activated VacA or control buffer or (p < 0.05) (Figure 4D). This suggests that *H. pylori* induction of apoptosis in *G. mellonella* hemocytes is, at least in part, dependent on the release of soluble virulence factor(s) including VacA cytotoxin.

## Discussion

In the present study, we provide evidence that the larva of the wax moth *G. mellonella* can be used as a new and simple infection model to study *H. pylori* virulence. We show that a panel of wild-type and mutant strains selectively defective in specific virulence factors are able to infect and kill *G. mellonella* larvae in a dose- and time-dependent fashion. All *H. pylori* strains analyzed are able to increase cell number by 1-log during infection of *G. mellonella* larvae, thus suggesting that *H. pylori* strains are able to survive and replicate in larvae. Our data also show that wild-type strain G27 is more virulent than wild-type strains 60190 and M5 and that *H. pylori* mutant strains defective in either VacA, CagA, CagE, *cag* PAI, or urease but not GGT-defective mutants, are less virulent than the respective parental strain. The increased ability to kill larvae of G27 strain compared with 60190 and M5 strains might be dependent on different genetic background and/or virulence factors different from VacA and CagA. That killing of larvae is dependent on the expression of a functional *cag* PAI and VacA cytotoxin is in accordance with previous data obtained in in vitro models showing that *H. pylori*-dependent epithelial cell damage and apoptosis of monocytes is dependent on VacA and *cag* PAI determinants [14]. Our data are also in agreement with those obtained in rodent models of *H. pylori* infection, in which inflammation and gastritis and apoptosis of monocytes and lymphocytes is dependent on the expression of both *cag* PAI and VacA [17,18]. While previous studies have shown that *H. pylori* GGT favours colonization of the gastric mucosa and more severe gastroduodenal diseases during infection in vivo [8,9], here we found no difference in killing of *G. mellonella* larvae between the GGT-defective isogenic mutant and its parental wild-type *H. pylori* strain. This discrepancy may depend on differences between *G. mellonella* and rodent models of infections and/or different experimental conditions.

We also evaluated the effect of *H. pylori* soluble/secreted virulence factors in *G. mellonella* larvae. In accordance with previous findings obtained in human and rodent models both in vitro and in vivo [13–18,41,44], we demonstrate that VacA, CagA and other *cag* PAI-encoded determinants are important soluble virulence factors of *H. pylori* strains. That soluble CagA mediates the killing of *G. mellonella* larvae is also in agreement with previous studies in a transgenic *Drosophila* model with inducible CagA expression which demonstrate that *H. pylori* CagA functions as a eukaryotic Grb2-associated binder (Gab) adaptor protein to activate the phosphatase SHP-2 and promote epithelial disruption or apoptosis through activation of the JNK signaling pathway [22,23].

Taken together, the data here presented demonstrate that *H. pylori* infection of *G. mellonella* larvae is a suitable model to study differences in virulence between strains. It is now well-known that *H. pylori* exhibits a high genetic and functional diversity in the *cag* PAI [5] as well as a high whole-genome variability among strains isolated from subjects either asymptomatic or affected by different gastroduodenal diseases [10–12]. In this respect, the infection of *G. mellonella* larvae may represent a useful model for the screening and the identification of virulence determinants in whole genome sequenced *H. pylori* strains.

Additional advantage provided by *G. mellonella* larvae infection model is the possibility to study the effect of strains and soluble virulence factors on the hemocytes, insect immune cells that are able to phagocyte bacterial and fungal cells [24] and to identify molecules responsible for immune evasion by *H. pylori*. Our data demonstrate that both *H. pylori* cells and soluble virulence factors induce apoptosis of insect hemocytes and that the effect is dependent on VacA and CagA and on the expression of a functional *cag* PAI. That CagA and *cag* PAI induce apoptosis of hemocytes is in agreement with previous studies showing that ectopic CagA expression induces apoptosis of epithelial cells of the *Drosophila* larval wing imaginal discs [23] and a functional *cag*PAI induces apoptosis of human monocytes [14]. Moreover, the effect of VacA on apoptosis of insect hemocytes is consistent with a previous study showing that VacA induces cell death in gastric epithelial cells [15,48] and inhibits dendritic cell maturation in neonatally infected mice [18]. Therefore, based on the data shown herein, we have identified specific bacterial virulence factors such as CagA, *cag* PAI components and VacA, which are able to evade host response of insect larvae.

A limitation of this study is that the strains used in our experiments differ in origins and lab passages. This might cause the various *H. pylori* mutants have additional uncharacterized differences compared to the single wildtype parental strain used. However, we were able to compare and duplicate the effect of mutants in identical genes, i.e. cagA and cagE, in two distinct genetic backgrounds, i.e. G27 strain versus 60190 strain. This issue might more properly be addressed by comparing the killing activity *in G. mellonella* larvae of several datasets of wild-type and isogenic mutants displaying different genetic backgrounds.

Based on the data shown herein, we hypothesize that CagA is injected into haemocytes via a type IV secretion system. Further studies will be necessary to demonstrate this hypothesis. The NFkB pathway, which has been demonstrated to be activated by CagA and *cag*PAI components during apoptosis of mammalian monocytes [2] and which is expressed in *G. mellonella* larvae [25], should be analyzed in hemocytes following *H. pylori* infection. In addition to the effects on hemocyte apoptosis, it should be interesting to study if *H. pylori* is able to colonize and induce damage to the midgut of *G. mellonella* larvae, as has been recently demonstrated for *C. jejuni* [36].

The above all experiments should be the matter of a future investigation.

## Conclusions

In conclusion, the model of *G. mellonella* larvae described herein represents a reliable and inexpensive model of *H. pylori* infection. Although the *G. mellonella* infection model cannot replace well-established and more “physiological” in vivo experimental models in the assessment of pathogenic mechanisms underlying *H. pylori*-related human diseases, it could be of use, and less expensive, for the evaluation of the effect of *H. pylori* virulence factors on specific cell functions.

This experimental model may reduce dependence on mammalian infection models and provide several applications for the *Helicobacter* research community such as the ability to distinguish between virulent and non-virulent *H. pylori* isolates, the identification of putative virulence genes through comparative genomics studies and the identification of novel molecular targets for antimicrobial therapy and vaccine development.

## Abbreviations

PAI: Pathogenicity island
GGT: Gamma-glutamyl transpeptidase
CagA: Cytotoxin-associated gene A
OD: Optical density
BCF: Broth culture filtrate
PBS: Phosphate-buffer saline solution
CFU: Colony-forming units
LD50: Lethal dose 50%
